# Polymorphism analysis of the *apxIA* gene of *Actinobacillus pleuropneumoniae* serovar 5 isolated in swine herds from Brazil

**DOI:** 10.1371/journal.pone.0208789

**Published:** 2018-12-18

**Authors:** Lucas Fernando dos Santos, Richard Costa Polveiro, Thalita Scatamburlo Moreira, Pedro Marcus Pereira Vidigal, Yung-Fu Chang, Maria Aparecida Scatamburlo Moreira

**Affiliations:** 1 Laboratory of Bacterial Diseases, Sector of Preventive Veterinary Medicine and Public Health, Veterinary Department, Universidade Federal de Viçosa, Viçosa, Minas Gerais, Brazil; 2 Microbiologia Veterinária Especial LTDA (Microvet), Viçosa, Minas Gerais, Brazil; 3 Núcleo de Análise de Biomoléculas (NuBioMol), Center of Biological Sciences, Universidade Federal de Viçosa, Viçosa, Minas Gerais, Brazil; 4 Department of Population Medicine and Diagnostic Sciences, College of Veterinary Medicine, Cornell University, Ithaca, New York, United States of America; National Cheng Kung University, TAIWAN

## Abstract

The bacterium *Actinobacillus pleuropneumoniae* is the etiological agent of Contagious Porcine Pleuropneumonia, a disease responsible for economic losses in the swine industry worldwide. *A*. *pleuropneumoniae* is capable of producing proteinaceous exotoxins responsible for inducing hemorrhagic lesions, one of which is ApxI. Few studies have conducted an in-depth evaluation of polymorphisms of the nucleotides that make up the *ApxI* toxin gene. Here we analyze the polymorphisms of the *apxIA* gene region of *A*. *pleuropneumoniae* serovar 5 isolated from swine in different regions in Brazil and report the results of molecular sequencing and phylogenetic analysis. Analysis of the *apxIA* gene in 60 isolates revealed the presence of genetic diversity and variability. The polymorphisms in the nucleotide sequences determined the grouping of the Brazilian sequences and five more sequences from the GenBank database into 14 different haplotypes, which formed three main groups and revealed the presence of mutations in the nucleotide sequences. The estimation of selection pressures suggests the occurrence of genetic variations by positive selective pressure on *A*. *pleuropneumoniae* in large groups of animals in relatively small spaces. These conditions presumably favor the horizontal dissemination of *apxIA* gene mutations within bacterial populations with host reservoirs. As a result, the same serovar can demonstrate different antigenic capacities due to mutations in the *apxIA* gene. These alterations in sequences of the *apxIA* gene could occur in other areas of countries with intense swine production, which could lead to differences in the pathogenicity and immunogenicity of each serovar and have implications for the clinical status or diagnosis of *A*. *pleuropneumoniae*.

## Introduction

*Actinobacillus pleuropneumoniae* is a bacterium belonging to the family Pasteurellaceae. It is a Gram-negative coccobacillus and the etiological agent of Contagious Porcine Pleuropneumonia, which is characterized by the development of necrotizing bronchopneumonia, hemorrhagic lesions in the lungs, cough and severe respiratory failure [1]. *A*. *pleuropneumoniae* is a highly contagious and sometimes lethal agent, being responsible for economic losses in pig farming around the world [2].

A number of virulence factors have been characterized in *A*. *pleuropneumoniae*, associated with the capsule, surface lipopolysaccharides, outer membrane proteins, biofilm formation and toxins of the RTX (repeats in toxin) family [3,4, 5–12, 13–15]. Apx cytotoxins are the main virulence factors related to the hemorrhagic lesions in Porcine Pleuropneumonia, being responsible for pore formation in cell membranes [16,17]. Apx exotoxins are highly immunogenic and induce a strong antibody response following *A*. *pleuropneumoniae* infection, and as such are considered to be ideal vaccine candidates [16].

Based on differences in capsular polysaccharides, 16 serovars have been described for *A*. *pleuropneumoniae* [18,19]; these secrete one or more of four RTX exotoxins [20]: ApxI, ApxII, ApxIII [16] and ApxIV [21]. Serovar 5 is the most common serovar in Brazil [22]. ApxI is a strongly hemolytic and cytotoxic toxin that is secreted by *A*. *pleuropneumonie* serovars 1, 5, 9, 10, 11 and 14. *A*. *pleuropneumonie* serovar 5 secretes ApxI, ApxII and ApxIV toxins, the latter being expressed in *vivo* [3,17,23,24]. The role of ApxIV toxin in *A*. *pleuropneumoniae* pathogenesis remains unclear [21] but it is essential for virulence [25,26]. Serovars that produce both ApxI and ApxII are responsible for the most severe Porcine Pleuropneumonia outbreaks worldwide [3,27,28].

**Fig 1:**
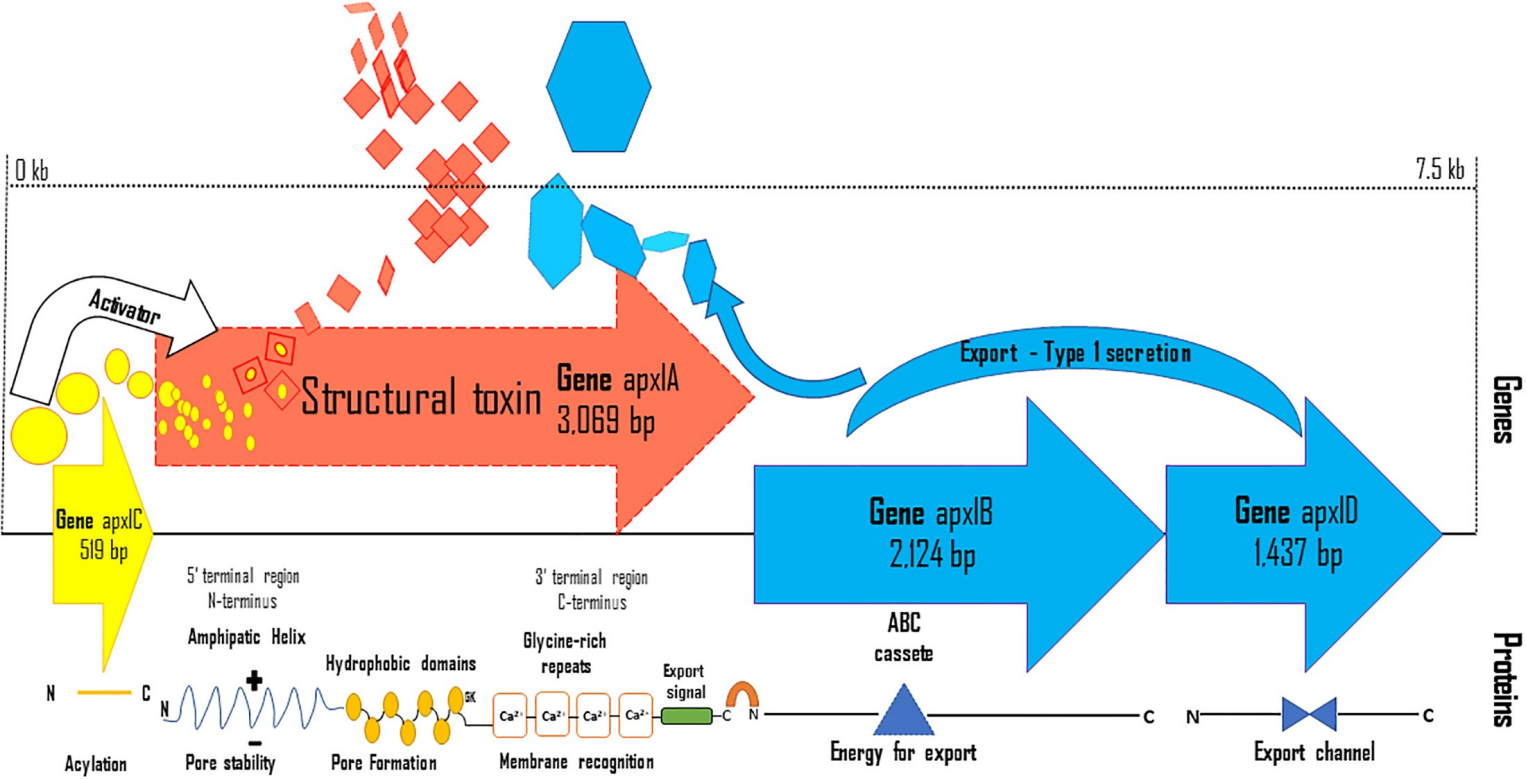
Structural and genetic organization of the ApxI toxin. Figure based on the structure and organization of the HlyA toxin of *Escherichia coli* RTX [31]. The genetic structure of *apxI* is shown at the top, represented by arrows demonstrating the direction of toxin transcription (*apxIC*, gene activator, *apxIA*, toxin structural gene, *apxIB* and *apxID*, toxin secretion genes), with the possible protein structure. The Rho-independent transcription termination signal between the *A* and *B* genes is indicated by a horseshoe. GK indicates the glycine-lysine acylation site (s). The ATP binding cassette on secretion protein B is abbreviated as ABC. N indicates the amino-terminal end and C the carboxy-terminal end of the peptides.

In general, those toxins are encoded in polycistronic operons that consist of contiguous genes *apxI*CABD, *apxIII*CABD and *apxII*CA [16,17,20], as shown Fig 1 (HlyA toxin from *Escherichia coli* RTX), that is, an a-hemolysin (HlyA) prototype of the RTX toxins produced by *Escherichia coli* [29]. In these operons, genes A and C are required for the production of structurally active toxin, gene A being responsible for encoding the toxin and gene C for encoding protein activation. Genes B and D are also required for toxin secretion. An exception to this is the apxIICA operon in *A*. *pleuropneumoniae*, which lacks the secretion genes B and D [16,17].

Few studies have conducted an in-depth evaluation of polymorphisms of the nucleotides that make up the *ApxI* toxin gene, or used bioinformatics tools such as bacterial haplotype reconstruction, which can aid in resolving mixed infections or in reconstructing the dynamics of evolving clonal populations of bacteria [30]. Therefore, in this study we used bioinformatics and phylogenetic analysis to survey the polymorphisms of the *apxIA* gene of *A*. *pleuropneumoniae* serovar 5 isolates from different pig-producing regions of Brazil. Our aim was to answer the following questions: (i) How conserved are the sequences of the *apxIA* gene among *A*. *pleuropneumoniae* isolates? (ii) Has the *apxIA* gene in *A*. *pleuropneumonie* accumulated mutations in different regions of Brazil? (iii) Where are these mutations predominant in the *apxIA* gene?

## Materials and methods

### Bacterial collection

Sixty-one isolates of *A*. *pleuropneumoniae* serovar 5 were analyzed in this study. The isolates were kindly provided by MicroVet Laboratory (Microbiologia Veterinária Especial Ltda, Viçosa/MG/Brazil) and were sampled between 2006 and 2011 from pig farms across seven Brazilian states: Espírito Santo (ES), Mato Grosso (MT), Mato Grosso do Sul (MS), Minas Gerais (MG), Paraná (PR), Rio Grande do Sul (RS), Santa Catarina (SC) and São Paulo (SP).

Of these samples, 41 had information on the type of swine breeding system, and 19 lacked this information. The farms from which samples were collected used two different breeding systems. In the Independent Breeding System, the farms operate a complete breeding cycle composed of gestation, farrowing, nursery, feeder and finishing sectors. In the Integrated Breeding System, the animals are moved between farms at different stages of development and the farms are basically composed of pig production and finishing units, which can house animals of different ages and origins.

The samples provided by MicroVet Laboratory were collected from pig tonsil scrapings from animals with clinical signs of Porcine Pleuropneumonia, using swabs placed in containers with Amies transport medium (Thermo Fisher Scientific Inc., Waltham, MA). According to MicroVet Laboratory, all animals were maintained in accordance with the standards of the Association for Assessment and Accreditation of Laboratory Animal Care International (AAALAC International).

The bacteria in the samples were first grown and identified on blood agar plates containing 5–10% sheep blood, incubated in an atmosphere of 5% CO_2_ at 37°C for 24 to 48 hours, and cross-inoculated with *Staphylococcus aureus* providing β-NAD (nicotinamide adenine dinucleotide). Afterwards, the isolates were identified, classified by biochemical tests, serovared and lyophilized.

The lyophilized samples were provided by MicroVet Laboratory and included isolates from different individual cases, not directly from outbreaks. To reactivate them, the isolates were rehydrated using 200 μL of autoclaved distilled water and then the colonies were inoculated on chocolate agar containing 50 μg/ml NAD (Sigma) and incubated at 37°C for 24 hours in a microaerophilic jar to obtain cell mass. The bacterial cell mass was collected with the aid of a loop and inoculated into a flask containing 30 mL of brain heart infusion medium (BHI). The bacteria were cultured at 37°C for 24 hours for subsequent genomic DNA extraction.

This study did not manipulate animals or conduct any experiment *in vivo*, and only received samples of lyophilized isolates that were kindly provided for analysis and research by the private company MicroVet Laboratory, which has no institutional relationship with the Universidade Federal de Viçosa.

### DNA extraction, PCR and sequencing

The genomic DNA of bacterial samples was extracted using the PureLink Genomic DNA kit (Invitrogen). Five mL of bacterial culture was centrifuged at 12,500 g for 10 minutes at room temperature, the supernatant was discarded and the obtained pellet was submitted to genomic DNA extraction according to the manufacturer’s instructions. Three pairs of primers were used in PCR to amplify the DNA samples (Table 1). The primer pair App5, which amplifies the *cps* gene, was used to confirm the presence of *A*. *pleuropneumoniae* serovar 5 in DNA samples [32]. The primer pairs ApxIA and pET apxIA were used to identify [33] and sequence the entire *apxIA* gene, respectively. The PCR runs were performed according to the conditions established by Ito [34] using a PxE Thermal Cycler (Thermo Scientific), and the isolate ATCC #33377 of *A*. *pleuropneumoniae* serovar 5 was used as a positive control. The PCR products were sequenced at Macrogen Inc. (South Korea).

**Table 1:** Primers used in the PCR for confirmation of *apxIA* gene amplification from *Actinobacillus pleuropneumoniae* serovar 5.

Gene	*Primers (5’-3’)*	Fragment Size (pb)	Reference
*cps* **(Confirmation)**	App5 F—TTT ATC ACT ATC ACC GTC CAC ACC T R—CAT TCG GGT CTT GTG GCT ACT AA	1100	Jessing et al,. [32].
*apx*IA **(Confirmation *apx*IA toxin)**	ApxIA F—ATC GAA GTA CAT CGC TCG GA R—CGC TAA TGC TAC GAC CGA AC	723	Rayamajhi et al,. [33].
*apx*IA **(Total amplification)**	pET apxIA F—CAC CAT GGC TAA CTC TCA GC R—TTA AGC AGA TTG TGT TAA ATA ATT AC	3069	Primers designed from the *apxIA* gene GenBank AF363361

Primer App5, a partial sequence of the serovar 5 serovar-specific *cps* gene, was used in the PCR technique to confirm the presence of *A*. *pleuropneumoniae* serovar 5 in the samples; *Apx*IA primers were used to confirm the presence of the *apxIA* gene and pET apxIA for total amplification of the *apxIA* gene.

### Sequencing analysis of *apxIA* gene

The sequencing chromatograms were analyzed and trimmed using Phred version 0.020425.c [35,36], selecting only the nucleotides sequenced with Phred scores greater > 20 (accuracy > 99). Then, contigs of the nucleotide sequences were assembled using the CLC Main Workbench 6.7.1 (CLC bio).

To complement the dataset, five *apxIA* gene sequences from *A*. *pleuropneumoniae* were downloaded from the GenBank database (https://www.ncbi.nlm.nih.gov/genbank/). The accession numbers for these sequences were as follows (with corresponding strains): NZ_CP029003.1 (4074, serovar 1), NC_009053.1 (L20, serovar 5b), ADOI00000000.1 (CVJ13261, serovar 9), ADOJ00000000 (D13039, serovar 10) and ADOK00000000.1 (56153, serovar 11). All sequences were aligned using MUSCLE version 3.8.31 with default settings [37] and the obtained alignment was manually inspected.

#### Setting the best-fit model of DNA evolution

Phylogenetic reconstructions by Bayesian Inference (BI) and Maximum Likelihood (ML) require the setting of a DNA evolution model to calculate the probabilities of nucleotide changes [38]. The “F81+I” was selected as the best evolution model of the *apxIA* gene by the jModeltest 2 program [39]. In the F81 model [40], all nucleotide substitutions are equally likely and the base frequencies are variable. The proportion of invariable sites (I) represents all alignment sites that do not change in the dataset.

#### The phylogenetic tree

To expedite the construction of the phylogenetic tree, the alignment was trimmed using Gblocks Server Version 0.91 [41] (using parameters for less stringent selection). The model of nucleotide substitution “F81+I” was chosen using the program jModelTest version 2.1.10 [39]. The phylogenetic tree was inferred by the Markov Chain Monte Carlo (MCMC) method using MrBayes v3.1.2 [42] and phylogenetic trees were calculated in two runs with 10,000,000 (ten million) generations and a sampling frequency of 10,000 (ten thousand). The parameter convergence was analyzed in Tracer version 1.6 (http://tree.bio.ed.ac.uk/software/tracer) and 10% of the trees generated were burned to produce the consensus tree. The heatmaps that accompany the phylogenetic trees were constructed by generating the distance matrix with Mega7 [43] software, with later use of the software R [44] together with the gplots package in the heatmap.2 function.

#### Genetic network

Genetic networks present an alternative view of genealogies represented by bifurcated structures of phylogenetic trees, and the possible dispersal routes of different toxin apxIA isolates in the Brazilian pork industry were predicted following the methodology described by Vidigal et al. [45]. To reconstruct the network, sequences of the *ApxIA* gene were grouped into 14 haplotypes using DnaSP v6 [46]. Following this, the network was constructed using the Network 4.6.1.0 (http://www.fluxus-technology.com) and the Median Joining algorithm (MJ) [47].

#### Estimation of selection pressures

The selective pressure on the 65 sequences of *apxIA* gene was evaluated according to the methodological approach used by Franzo et al. [48], with minor modifications to detect the sites of nucleotides and amino acids under selection. This approach involved the following methods: SLAC (Single-Likelihood Ancestor Counting), FEL (Fixed Effects Likelihood), FUBAR (Fast, Unconstrained Bayesian Approximation) and MEME (Mixed Effects Model of Evolution) [49–51], found on DataMonkey 2.0 [52], a web-server of the HyPhy [53]. The web application DataMonkey only calculates selection from the phased haplotype data. FEL, SILAC and MEME use a maximum-likelihood (ML) approach, and FUBAR uses a Bayesian approach. These methods are based on the estimation of the difference between non-synonymous and synonymous substitution rates (dN-dS), which allowed the estimation of pervasive and episodic diversifying/purifying selection [48].

The significance value was set to p < 0.05 for the FEL and MEME methods and to p < 0.05 for SLAC. The results of FUBAR indicate positive selection using posterior probabilities with values of 0–1 and not *p*-values, and were accepted when the posterior probability was greater than 0.9 FUBAR. Sites were assumed to be under diversifying selection when detected by MEME, and also able to model episodic diversifying selection, or by at least two other methods [48]. The SLAC claims to be more conservative [54] and tends to underestimate the substitution rates [51]. Furthermore, to estimate genetic distances a plot analysis was conducted in SSE version 1.3 [55], using the *p*-distance model for all sites with a sliding window of 200 bp and step size of 20 bp.

#### Nucleotide sequence accession numbers

The 60 complete coding sequences of the *ApxIA* gene were submitted to GenBank database as haplotypes under accession numbers: KY468982 (H1), KY468983 (H2), KY468984 (H3), KY468985 (H4), KY468986 (H5), KY468987 (H6), KY468988 (H7), KY468989 (H8), KY468990 (H9), KY468991 (H10), KY468992 (H11), KY468993 (H12), KY468994 (H13) and KY468995 (H14) (Table 2).

**Table 2:** Diversity of haplotypes and phylogenetic groups in the Independent Breeding and Integrated Breeding Systems.

Systems breeding swine	Years ^a^	Swine breeders ^b^	Isolates ^c^	Haplotypes ^d^	States ^e^	Phylogenetic clade ^f^
**Independent Breeding**	2006	B	426	H1	SC	green
		C	431			
		C	430	H2		
		D	439	H3	SP	red
		D	461	H5		green
		I	449	H4	ES	red
	2007	G	523	H3	SC	
		J	491	H6	PR	green
		K	510	H3	SC	red
		K	511	H7		
		L	509	H3		
		M	524			
	2009	E	725	H1	RS	green
		F	767			
		N	770		SC	
		N	769	H6		
		O	791	H9	MS	red
	2010	H	888	H1	MG	green
		A	867	H13	RS	red
		P	861	H11	SC	
**Integrated Breeding**	2008	Q	643	H1	SC	green
			674			
			680			
			700			
			672	H8		
	2009		766	H1		
			758			
			745	H6		
			761	H3		red
			760			
	2010		915	H1		green
			919			
			920			
			891	H3		red
			893			
			912			
			914			
			857	H10		
			913	H11		
			862	H12		
			928	H14		green

^a^ Year in which the isolate was collected

^b^ Property at which the sample was collected;

^c^ Identification of isolates in laboratory;

^d^ Groups of haplotypes;

^e^ Brazilian state of origin of the isolates that determine the haplotype;

^f^ Phylogenetic clade determined in the consensus tree.

## Results

Of the 61 isolates, only one did not amplify for serovar 5 in the reaction with *cps* primers and thus was not used in subsequent studies. The remaining 60 isolates were confirmed as *A*. *pleuropneumoniae* serovar 5. The sequences obtained corresponding to the *apxIA* gene of the isolates were divided into 14 haplotypes and represented 3066 bp, corresponding to the complete sequence of the *apxIA* gene [56].

**Fig 2:**
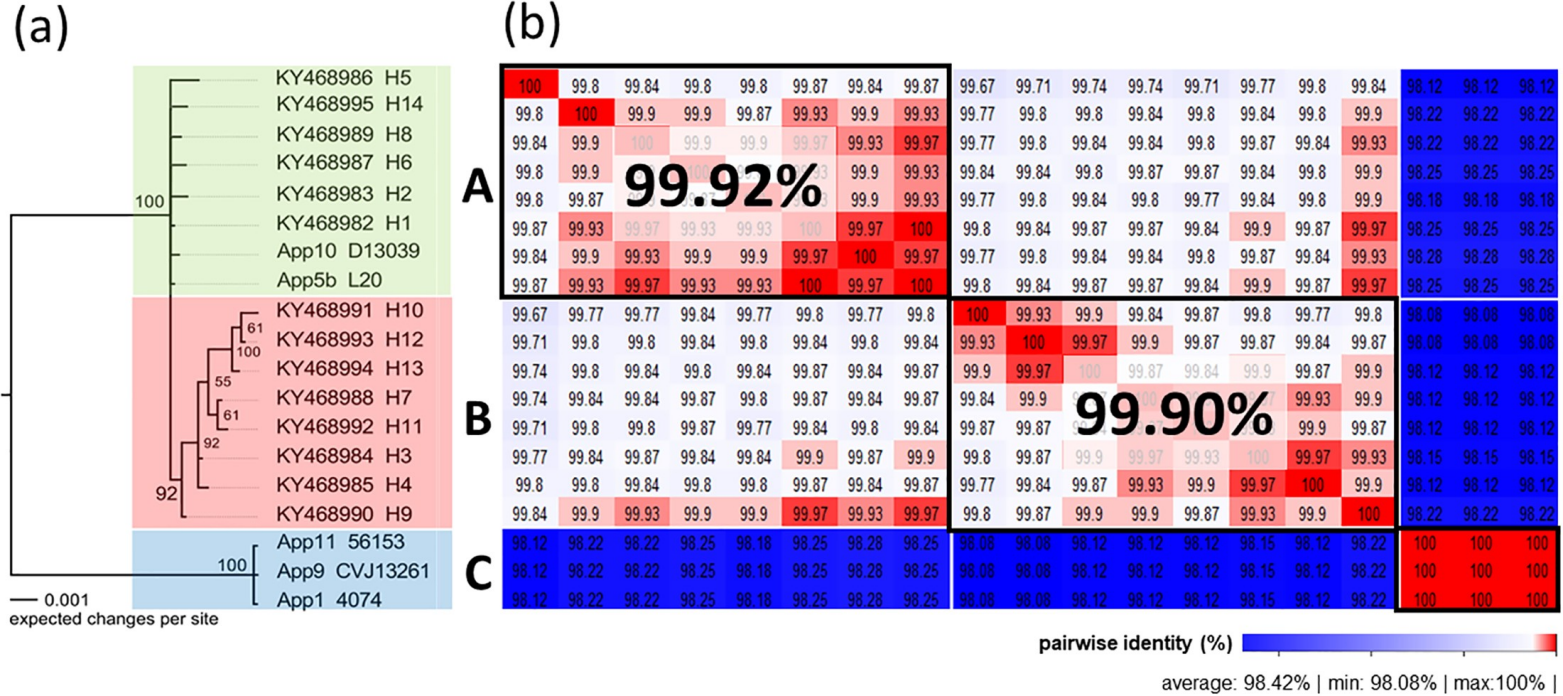
Consensus tree (2a) and the heatmap distance matrix of pairwise comparisons (2b). **a** The phylogenetic tree (Fig 2a) obtained by the Bayesian analysis of the 60 sequences of the *apxIA* gene of *A*. *pleuropneumoniae* serovar 5 isolates of swine from different Brazilian states, plus five isolates from the GenBank database. The posterior probability values (PP) (expressed as percentages) calculated using the best trees found by MrBayes are shown beside each node. Clades were divided by identity into three monophyletic clades: green clade (A), red clade (B), and blue clade (C). **b** The heatmap distance matrix (Fig 2b) represents high nucleotide identity in red and low identity in blue, and together with the phylogenetic tree identifies the clades in groups of major/minor identity, surrounded by squares with black edges, indicating the actual percentage of identity.

The phylogenetic tree (Fig 2a) is a consensus tree that, together with the heatmap distance matrix of pairwise comparisons (Fig 2b), groups the Brazilian isolates and compares these with the sequences obtained in the GenBank database according to their identity. The phylogenetic tree (Fig 2a), which comprises 60 Brazilian sequences, along with the five GenBank sequences (NZ_CP029003.1, NC_009053.1, ADOI00000000.1, ADOJ00000000 and ADOK00000000.1), shows three distinct monophyletic clades: green (A), red (B) and blue (C). Clade A consists of 40 Brazilian sequences and two sequences deposited and publicly available in GenBank (*A*. *pleuropneumoniae* serovar 10 and 5b), whereas clade B consists of 20 Brazilian sequences. Clade C is composed of three sequences (*A*. *pleuropneumoniae* serovar 1, 9, 11) available in the GenBank database.

The heatmap distance matrix (Fig 2b) represents the highest nucleotide identity in red and the lowest in blue. The identity was 99.92% on average, which means high conservation between sequences of the *apxIA* gene of *A*. *pleuropneumoniae* isolates. The haplotypes H1, H2, H5, H6, H8, and H14 and the sequences of *A*. *pleuropneumoniae* 10 and 5b demonstrated more than 99.92% identity, represented by the green clade (A). The H10, H12, H13, H7, H11, H3, H4, and H9 haplotypes of the red clade (B) showed 99.90% identity, whereas the sequences of *A*. *pleuropneumoniae* 11, 9 and 1 of the blue clade (C) presented high identity, but lower identity in relation to A and B. The complete list of *apxIA* gene sequences separated by clades and also by haplotypes used in this study is available in S1 Table.

Table 2 demonstrates the diversity of haplotypes based on 41 sequences from the same or different breeding farms. In the Independent Breeding System, different haplotypes within the same phylogenetic clade and different phylogenetic clades were described at the same property. In swine breeder C in the state of SC, two different haplotypes, i.e. H1 and H2, were found, both of which were isolated in 2006 and belong to the green clade (A) (Fig 2a). In swine breeder D in the state of SP state, the H3 and H5 haplotypes were found in the same year (2006), with H3 belonging to clade A and H5 to the paraphyletic red clade (B). Swine breeder K in the state of SC presented a diversity of haplotypes in 2007, with haplotypes H3 and H7 belonging to group 2 being found. Swine breeder N, also from SC state, presented two haplotypes in 2009, i.e. H1 and H6, both belonging to the green clade (A). Another 19 sequences that were not cataloged for the breeding system are listed in S2 Table, and of these, 18 were assigned as belonging to the green clade (A) and H1, and one of them as belonging to the red clade (B) and H3.

Eight different haplotypes were identified in 21 isolates from swine breeder Q in SC state, with nine isolates belonging to haplotype H1, six isolates to haplotype H3, and one isolate for each of the other haplotypes (H6, H8, H10, H11, H12, H14). In 2008, haplotypes H1 and H8 were found, both belonging to the green clade. In 2009, three different haplotypes were found, i.e. H1 and H6 belonging to the green clade (A) and H3 belonging to the red clade (B). A greater diversity of haplotypes was found in 2010, with haplotypes H1 and H14 belonging to the green clade, and haplotypes H3, H10, H11 and H12 belonging to the red clade (B).

Table 2 shows the distribution of the six haplotypes found within 21 samples from facilities using the Independent Breeding System alone (H2, H4, H5, H7, H9, H13) and the four different haplotypes found among 21 samples from facilities using the Integrated Breeding System alone (H8, H10, H12, H14). Four haplotypes (H1, H3, H6, H11) were found in swine bred using both the Integrated and Independent systems.

**Fig 3:**
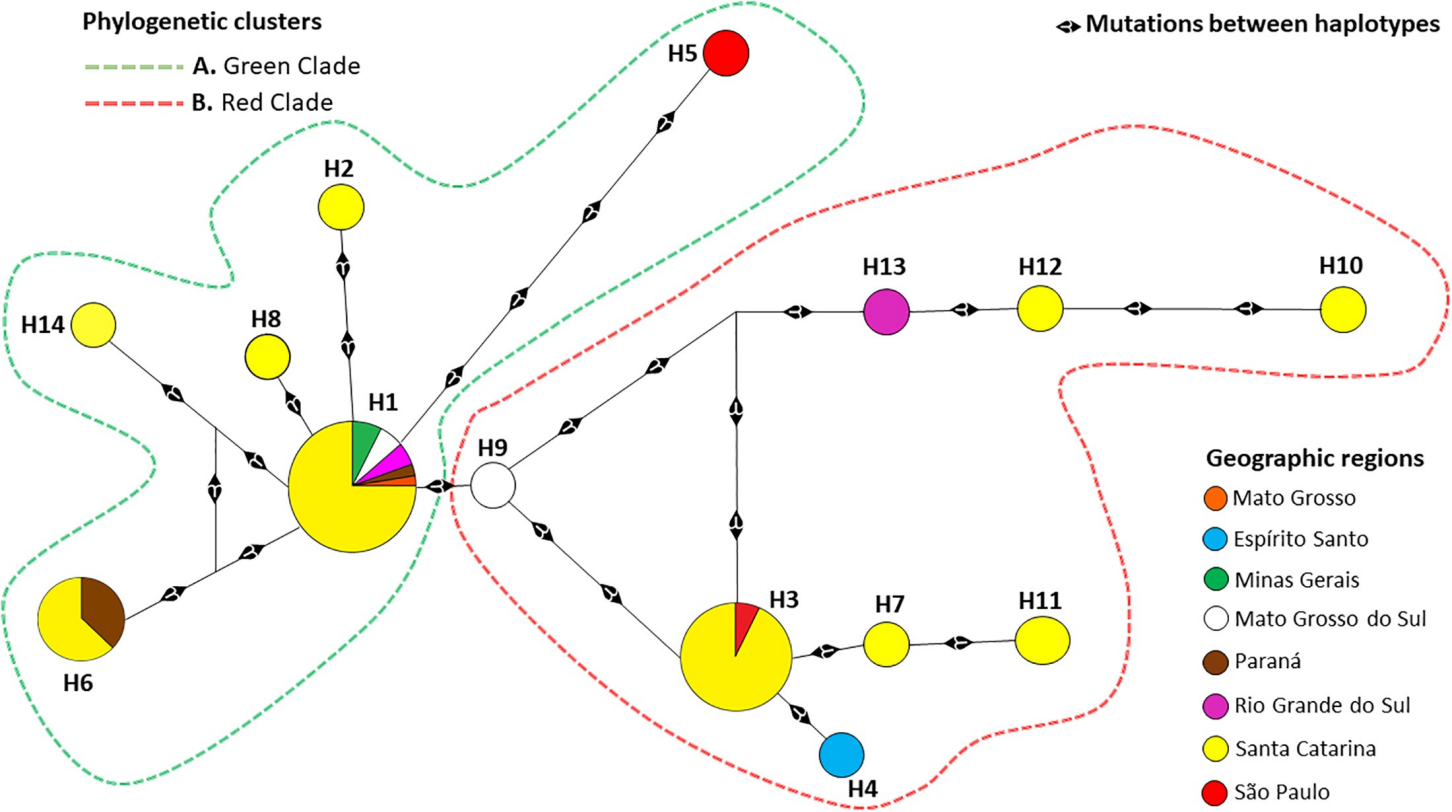
Genetic network of the 60 sequences of samples of *apxIA* gene in Brazil. The haplotypes are colored according to the geographical state in which the sample was collected. The size of the circle halo is proportional to the relative frequency or number of samples in the group of haplotypes. The black arrows indicate the number of mutations that differentiate one haplotype from the nearest haplotype.

Table 3 was constructed in association with Fig 3, and shows the haplotypes published in GenBank, haplotype code, and the number of samples recorded per haplotype. The H1 haplotype was the most frequent (33/60), being found in six states: MG, MS, MT, RS, PR, and SC. The second most frequent haplotype was haplotype H3 (12/60), recorded in the states of SC and SP. The H6 haplotype was found in the states of SC, SP and PR, presenting a frequency of 3/60. The H11 haplotype (2/60) was only observed in SC state. The other haplotypes, from different states, recorded only one *A*. *pleuropneumoniae* serovar 5 isolate.

**Table 3:** Identification of haplotypes in association with their frequency.

Ids ^a^	Coding of Haplotype	Frequency ^b^	Indep. Bred ^c^	Integ. Bred ^d^	System not cat. ^e^
KY468982 ^cd^	H1 ^cd^	33	6	9	18
KY468983	H2^c^	1	1	0	0
KY468984 ^cd^	H3 ^cd^	12	5	6	1
KY468985	H4 ^c^	1	1	0	
KY468986	H5 ^c^	1	1	0	0
KY468987 ^cd^	H6 ^cd^	3	2	1	0
KY468988	H7 ^c^	1	1	0	0
KY468989	H8^d^	1	0	1	0
KY468990	H9 ^c^	1	1	0	0
KY468991	H10 ^d^	1	0	1	0
KY468992 ^cd^	H11 ^cd^	2	1	1	0
KY468993	H12 ^d^	1	0	1	0
KY468994	H13 ^c^	1	1	0	0
KY468995	H14 ^d^	1	0	1	0
Total samples		60	20	21	19

^a^ GenBank identity;

^b^ Number of isolates included in a given haplotype group;

^c^ Independent Breeding;

^d^ Integrated Breeding;

^e^ System not cataloged;

^cd^ Found in both systems.

Fig 3 illustrates the clustering between 14 haplotypes (groups of sequences with 100% identity after trimming) and demonstrates the dispersion of different mutations within the sequences of the *apxIA* gene in states of Brazil. These haplotypes correspond to groups of 60 isolates with the *apxIA* gene sharing identical sequences. Fig 3 shows the presence of 14 haplotypes with the geographical origin of the sample for each Brazilian state shown in different colors. The dotted lines surrounding each group indicate the grouping of the haplotypes in the monophyletic clades, as indicated previously in Fig 2a, thus adding information to the phylogenetic analysis. Information on the nucleotide positions at which the mutations occurred can be found in S1 Fig.

Most of the haplotypes were separated by one or a few mutations, except haplotype H5, which was separated by four mutations in the genetic network. H3 and H9 were separated from other haplotypes (H3, H13 and H1) by different numbers of mutations. In addition, the genetic network showed that two haplotypes (H1 and H3) comprised a large number of isolates of the *apxIA* gene from several states of Brazil (SC, MG, MS, RS, PR, MT, SP). H5 presented low similarity in relation to the other haplotypes in its green clade (A) due to its relatively large number of mutations in relation to the other haplotypes. The state of SC displayed the greatest variability in haplotypes.

The H1 haplotype appears to be a possible ancestor of haplotypes H2 (Santa Catarina), H5 (São Paulo), H6 (Santa Catarina, Paraná), H8 (Santa Catarina), and H14 (Santa Catarina), and haplotype H3 (Santa Catarina, São Paulo) could be a possible ancestor of H4 (Espirito Santo) and H7 (Santa Catarina).

**Fig 4:**
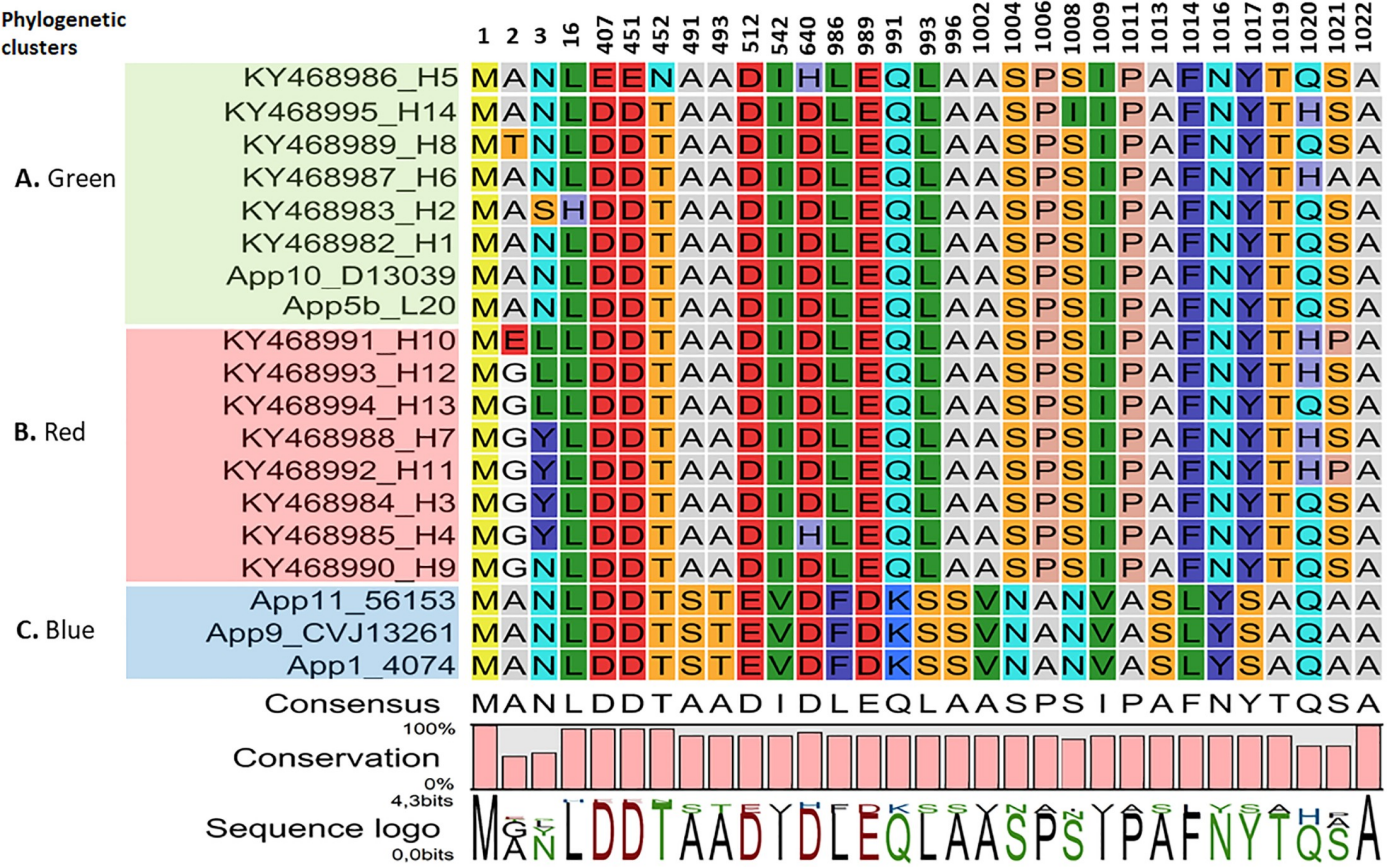
Polymorphisms identified in the amino acid sequence of ApxIA protein. The peptides were grouped according to the haplotypes and phylogenetic clades determined in Fig 2a. The figure presents the localization of amino acid mutations between the first (1) and the final (1022) amino acid of the ApxIA toxin of Brazilian isolates of *A*. *pleuropneumoniae* serovar 5 and GenBank serovars 11, 10, 9, 5 and 1.

The differences in the primary structure given by the amino acid sequence of the Brazilian sequences grouped into 14 haplotypes and the five sequences from GenBank are shown in Fig 4. The isolates were grouped according to the arrangement of the clades present in Fig 2a. Twenty-nine alterations were predicted among the 65 sequences analyzed and no alterations were found for the remainder of the sites in the sequences. The differences between the green and red clades were mainly at sites 2, 3 and 1020 and 1021 of the peptides. The blue clade, represented by sequences of *A*. *pleuropneumonie* serovar 11, 9 and 1 of the *apx*IA gene, presented a high number of alterations in its primary structure and in almost the same positions as the other haplotypes. Position 2 in the green clade was occupied by alanine (A) while in the red clade it was occupied by glycine (G). At site 3 of the protein, the green clade has an asparagine residue (N), while H2 apxIA has a serine residue (S). The red clade is composed of the amino acid tyrosine (Y) to a large degree, and also has a leucine (L) residue. The amino acid alterations found did not cause major changes in polarity related to hydrophobic or polar amino acids.

The results of the tests used to verify possible positive evolution/diversification or negative/purification pressure events on the apxIA toxin (amino acid codons) can be found in S3 Table. The results indicated two codons under positive evolutionary pressure or selection of diversification, and a further 12 codons under negative evolutionary pressure or purification selection. The MEME test found two codons with a *p*-value of up to <0.05, indicating the possibility of episodic positive/diversifying selection. The FEL test with a p-value of <0.05 found a codon under episodic positive/diversifying selection and two codons under pervasive negative/purifying selection. The FUBAR test with a probability value greater than 0.9 indicated a codon under episodic positive/diversifying selection and 11 codons under pervasive negative/purifying selection. Tests using the SILAC methodology did not return significant results with a p-value < 0.05. Codon number 3 presented results indicative of episodic positive/diversifying selection in three tests.

In S2 Fig, constructed using the ESS program, it is possible to check the points at which the *apxIA* gene mutations were most evident among the 3066 nucleotides and 1022 amino acids responsible for the apxIA toxin structure, and also how these changes can be differentiated into synonymous and non-synonymous mutations between the green (A), red (B) and blue (C) clades. Notably, the main polymorphisms occur at the C-terminal end, in the middle of the gene and at the N-terminal, when compared to Fig 1. However, the mid-gene mutations were predominantly synonymous and the mutations at the extremities were generally non-synonymous. The blue x green clade (pink line) and the blue clade x red clade (cyan line) are superimposed on the graph. This overlap is due to the fact that the blue clade (A) formed by *A*. *pleuropneumoniae serovar* 11, 9 and 1 has more mutations than the other clades, with marked nucleotide differences when compared to the green (A) and red (B) clades, which show greater genetic identity as demonstrated in Fig 2.

## Discussion

The present study investigated the variability of the *ApxIA* gene present in the ApxI operon (Fig 1), which is responsible for the structural region of the ApxIA toxin in *A*. *pleuropneumoniae* serovar 5, and has already been confirmed as an important inducer of immune responses [57–59]. The phylogenetic analysis of the *apxIA* gene in the 60 isolates and five other sequences deposited in GenBank (Fig 2) revealed the presence of mutations between the nucleotide sequences (Figs 3 and 4, S1 Fig and S3 Table). From this it was possible to group the Brazilian sequences into 14 different haplotypes and the other five sequences into three dominant groups named the green (A), red (B), blue (C) clades (Fig 2), distributed as a series of genetically identical sequences in Fig 3 with genetic network. The *apxIA* gene of *A*. *pleuropneumoniae* serovar 5 appeared similar to that of serovar 10, but different from serovar 11, 9 and 1.

The separation of the sequences into three monophyletic clades in Fig 2 suggests a modification in the *apxIA* gene, due to a possible adaptation of the *A*. *pleuropneumoniae* serovar 5 circulating in Brazil, indicating that the bacteria may have been subjected to positive selection pressure resulting from intensive swine production methods [60]. Xu et al. [61] suggested that genetic variations caused by positive selective pressure (S3 Table) in *A*. *pleuropneumoniae* can influence population diversification and adaptation, including the mechanisms of immune evasion and the host–pathogen interaction. In addition, the data suggest that the polymorphism of the *apxIA* gene is subjected to diversifying selection and purifying selection in different codons of amino acids (S2 Fig and S3 Table).

Proteins such as the apxIA toxin retain their surface exposure to the host immune system [62] and for that reason are subject to positive selection [63]. S3 Table and S2 Fig illustrate the occurrence of a large number of codons under purifying selection and the high number of synonymous mutations, respectively. In a study by Hughes [64], purifying selection was not found in the case of non-synonymous mutations but did occur in synonymous mutations, since non-synonymous mutations result in a slightly diminished level of activity for this protein.

Satomi Kuchiishi et al. [22] reported on the importance of the epidemiology of the serovars in Brazil, and the differences in the dynamics of *A*. *pleuropneumoniae* between farms, which raise questions about the modifications and adaptations of *A*. *pleuropneumoniae*, as described in this study. The presence of variations in the *apxIA* gene, as previously suggested by Nagai et al. [65], indicates that the *apxIA* gene could be divided into three groups or clades (A, B, C) (shown in Fig 2). However, the factors responsible for generating the appearance and spread of *apxIA* mutations in subpopulations/serovars of *A*. *pleuropneumoniae* remain to be determined

The data in Fig 3 that demonstrate the circulation of different haplotypes within a single state in Brazil are consistent with other studies, which have reported multiple serovars and strain types circulating within a population in an area that is geographically restricted [66–69]. In the genetic network (Fig 3), the central haplotypes are probable ancestors and it is possible to reconstruct reticulations that cannot be represented in a phylogenetic tree [45,70,71]. These reticulations reflect constraints such as homoplasy, parallel, or convergent evolutionary paths [70]. This demonstrates that the number of distinct haplotypes of *A*. *pleuropneumoniae* (Fig 3), reported in study of *Salmonella enterica* serovar Typhi [72], can circulate in a relatively small geographical area or are currently circulating in different regions of the same continent, within states or countries that possess large-scale pig farming operations such as those mentioned in this study.

Of the 14 haplotypes identified in this study, Independent Breeding facilities presented six rare haplotypes, while Integrated Breeding facilities presented four (Table 2 and Fig 3). These results, however, may reflect an inadvertent bias due to the small sample size obtained from Integrated Breeding facilities. Companies that implement Integrated Breeding or Vertical Integration Systems control management practices, nutrition, and technical assistance, although the animals are distributed to different farms to finish growing and fattening, which can lead to the confinement of swine of different origins in one location. Thereby, the chances of distributing subclinically infected pigs within Integrated Breeding Systems are high, facilitating the (unintended) spread of *A*. *pleuropneumoniae* [73–75].

Integrated Breeding or Vertical Integration presumably promotes the mixing of subclinically infected pig subpopulations; this favors the dissemination of mutations in the *apxIA* gene of *A*. *pleuropneumoniae*. In other studies, with agents such as *Salmonella* sp. serovars [76], it was concluded that the Vertical Integration System does not necessarily achieve superior control of *Salmonella* sp. in the swine production system.

While multiple serovars may be present on a single farm, and even with a single host [75], a given serovar can carry different mutations in the *apxIA* gene, the products of which display different antigenic characteristics. Independent Breeding System facilities feature complete breeding cycles on a single farm. This may result in differences in the *apxIA* haplotype profiles found at different facilities. Nonetheless, if haplotypes are adapted to particular environments, then the combination of hitch-hiking events and low levels of recombination would explain the observed distribution of genetic variability seen in this system [77].

In swine production, especially in intensive production templates such as Independent Breeding or Integrated Breeding Systems, animals are typically housed in large groups in relatively small spaces, thereby providing ideal conditions for maintaining pathogen populations for extended periods of time [60]. This system places haplotypes under severe selection pressures, in which mutation rates and population size become sufficiently high to build up genetic variation in the bacterial population [78,79], resulting in a mixture of closely related haplotypes (or quasispecies) [30]. As a result, the bacterial population is usually not genetically uniform [80] in these types of production environments.

Previous studies using different *A*. *pleuropneumoniae* serovars have shown that there are significant variations in the genome between *apx* genes, which implies differences in the pathogenicity and immunogenicity of each serovar [5,65,81]. In addition, reported differences in the virulence of certain isolates of the same serovar could be due, at least in part, to the lack of production of one of the Apx toxins through deletions, point mutations or insertions of a transposon such as *ISApl1* [75]. Based on what is shown in Fig 4 and S3 Table, the amino acids for codon 3 (Methionine) at the 5’ end or the N-terminal region (Fig 1) and the 3’ end codon 1022 (Arginine) at the carboxyl or C-terminal, were likely the peptides that represented the greatest differences between haplotypes. Other important modifications were noted in the middle part of the *apxIA* gene as shown in Fig 4, S2 Fig and S3 Table. These were similar to the observations made by Nagai et al. [65] in the C-terminal and middle part of the *apxIA* gene. The alterations observed in the *apxIA* gene sequence and primary amino acid sequence of the *apxIA* protein may lead to changes in the secondary structure of the proteins (Fig 4) of *A*. *pleuropneumoniae*, as well as the structure of the protein toxin, but it is still unclear whether these changes lead to functional changes in the apxIA toxin.

Thereby, it is likely that there are differences in the secondary structures of the ApxIA toxin, although the central portions of the *apxIA* gene were shown to be highly conserved. The N-terminal and C-terminal regions, by contrast, appeared to contain the bulk of mutations observed in the sequence of this gene; this indicates that the various mutations seen in the termini of *apxIA* do not have a drastic effect on the activities of the ApxIA toxin product (Fig 1), as demonstrated in Fig 4 (conservation plot) and S2 Fig. The less well-conserved N- and C-terminal portions of the *apxIA* gene form the primary basis for classification of the various *A*. *pleuropneumoniae* isolates into 14 haplotypes and three GenBank groups (Fig 2). Furthermore, these data provide a new understanding of the modifications to the ApxI toxin, suggesting possible modifications to the toxin structure, which needs to be evaluated *in vivo*.

## Conclusions

In conclusion, important sequence variations in the *apxIA* gene of *A*. *pleuropneumoniae* serovar 5 were found in several swine breeding farms in Brazil. These sequence variations were the basis for dividing 60 samples and five other sequence groups of serovar 1, 9, 10 and 11 into three phylogenetically different groups and 14 haplotypes. These divisions into groups or clades may have occurred as a result of host breeding processes selecting mutations in the *apxIA* gene (haplotype), ensuring the spread these mutations and thereby generating a variety of different *A*. *pleuropneumoniae* serovars and clades residing in host populations. However, future in *vivo* studies will be necessary to evaluate the different immune responses to these haplotypes, due to a possible significant alteration in the toxin ApxIA protein. This study contributes to our knowledge of the epidemiology of *A*. *pleuropneumoniae*, providing information about the genetic diversity of an important toxin of this porcine pathogen.

## Supporting information

S1 Fig The positions of the nucleotides where the mutations occurred within the apxIA gene sequences in the Brazilian states. The numbering in red represents the position of the nucleotide in which nucleotide alterations occurred between the different haplotypes. Built from DnaSP v6 Network 4.6.1.0 and programs.
(PDF)

S2 Fig Plot of divergence of the *apxIA* gene sequences presented by means of the distance method *p*, calculated between and among the groups with Green (A), Red (B) and Blue (C) clades. Consisting of 14 haplotypes of *A*. *pleuropneumoniae* serovar 5 and five serovar 11, 10, 9, 5b and 1 sequences from the GenBank database. The vertical and horizontal axes indicate the pairwise distance and the genome position (bp) of the alignment, respectively. **a** The divergence between and among all 3066 bp of clades. **b** The divergence between and among 1022 amino acids of clades. **c** The difference between and among substitutions synonymous for all 3066 pb of clades. **d** The difference between and among substitutions non-synonymous for all 3066 bp of clades.
(PDF)

S1 Table The complete list of *apxIA* gene sequences separated by clades according to phylogenetic relationships. Sequences are divided between the clades green (A), red (B) and blue (C), separated by the number of sequences for each H1—H14 haplotype. Sequences related to the GenBank database are divided between the clades according to the separation made by phylogenetic analyzes.
(PDF)

S2 Table Sequences that were not cataloged for the breeding system. ^a^ Year in which the isolate was collected;^b^ Property in which the sample was collected;^c^ Identification of isolates in laboratory (isolates were shortened in two digits after the dash);^d^ Groups of haplotypes;^e^ Brazilian state of origin of the isolates that determine the haplotype;^f^ Phylogenetic clade determined in the consensus tree.
(PDF)

S3 Table Table reporting sites detected under statistically significant diversifying selection positive and negative (gray shadow) by three different methods. α = Synonymous substitutions rate at a site. β^+^ = Non-Synonymous substitutions rate at a site. [α<β] = Posterior probability of positive selection at a site. [α>β] = Posterior probability of negative selection at a site. Significance values (p-value/Posterior probability) are in bold.
(PDF)
