# Supplementary figures and images for: Polymorphism analysis of the apxIA gene of Actinobacillus pleuropneumoniae serovar 5 isolated in swine herds from Brazil
Source: PLoS One. 2018 Dec 18;13(12):e0208789. doi: 10.1371/journal.pone.0208789 (PMC6298653; doi:10.1371/journal.pone.0208789)

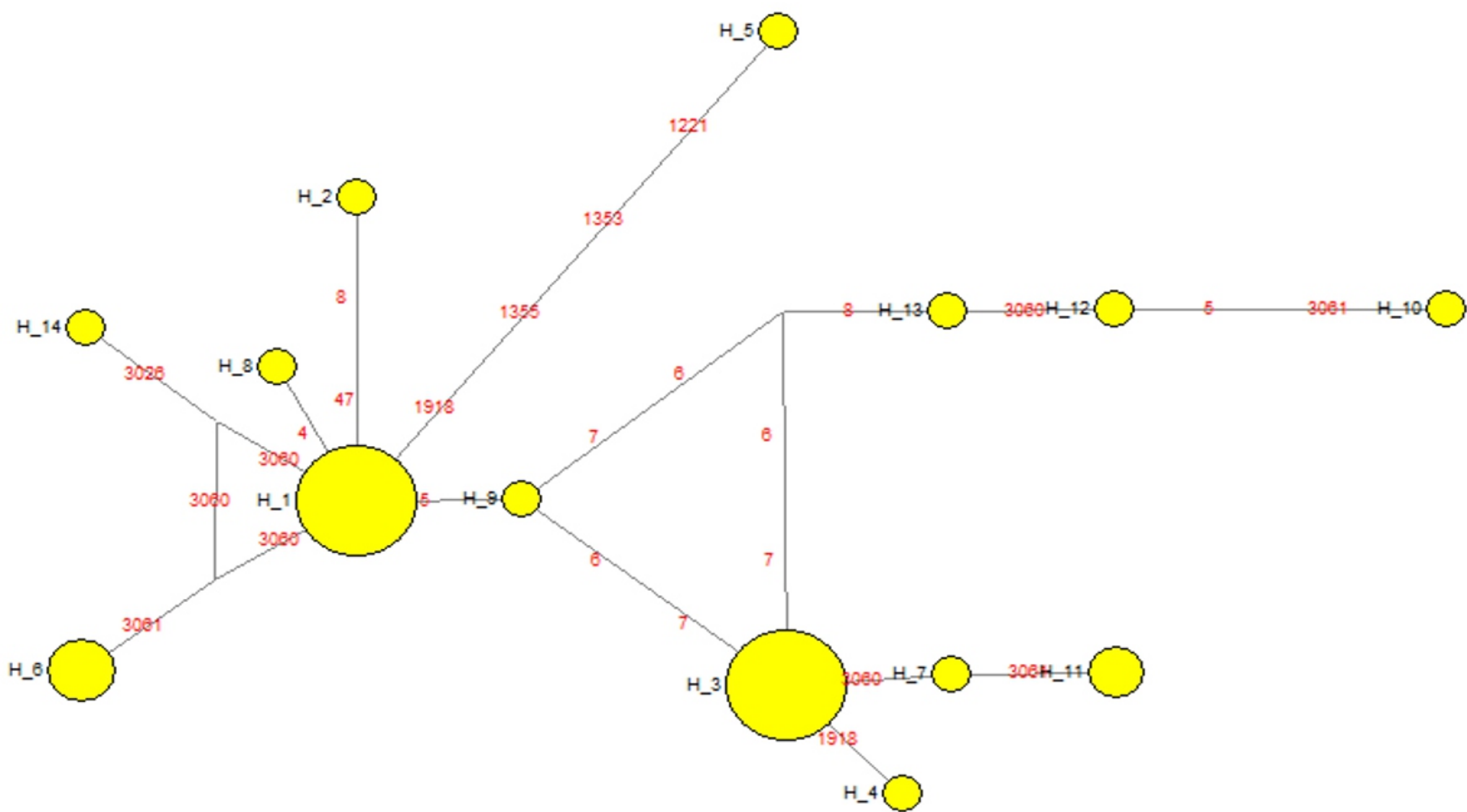

Supplement: S1 Fig — The numbering in red represents the position of the nucleotide in which nucleotide alterations occurred between the different haplotypes. Built from DnaSP v6 Network 4.6.1.0 and programs. (PDF) [file pone.0208789.s001.pdf]
