# Supplementary material for: Polymorphism analysis of the apxIA gene of Actinobacillus pleuropneumoniae serovar 5 isolated in swine herds from Brazil
Source: PLoS One. 2018 Dec 18;13(12):e0208789. doi: 10.1371/journal.pone.0208789 (PMC6298653; doi:10.1371/journal.pone.0208789)
Supplement: S2 Fig — Plot of divergence of the apxIA gene sequences presented by means of the distance method p, calculated between and among the groups with Green (A), Red (B) and Blue (C) clades. Consisting of 14 haplotypes of A. pleuropneumoniae serovar 5 and five serovar 11, 10, 9, 5b and 1 sequences from the GenBank database. The vertical and horizontal axes indicate the pairwise distance and the genome position (bp) of the alignment, respectively. a The divergence between and among all 3066 bp of clades. b The divergence between and among 1022 amino acids of clades. c The difference between and among substitutions synonymous for all 3066 pb of clades. d The difference between and among substitutions non-synonymous for all 3066 bp of clades. (PDF) [file pone.0208789.s002.pdf]

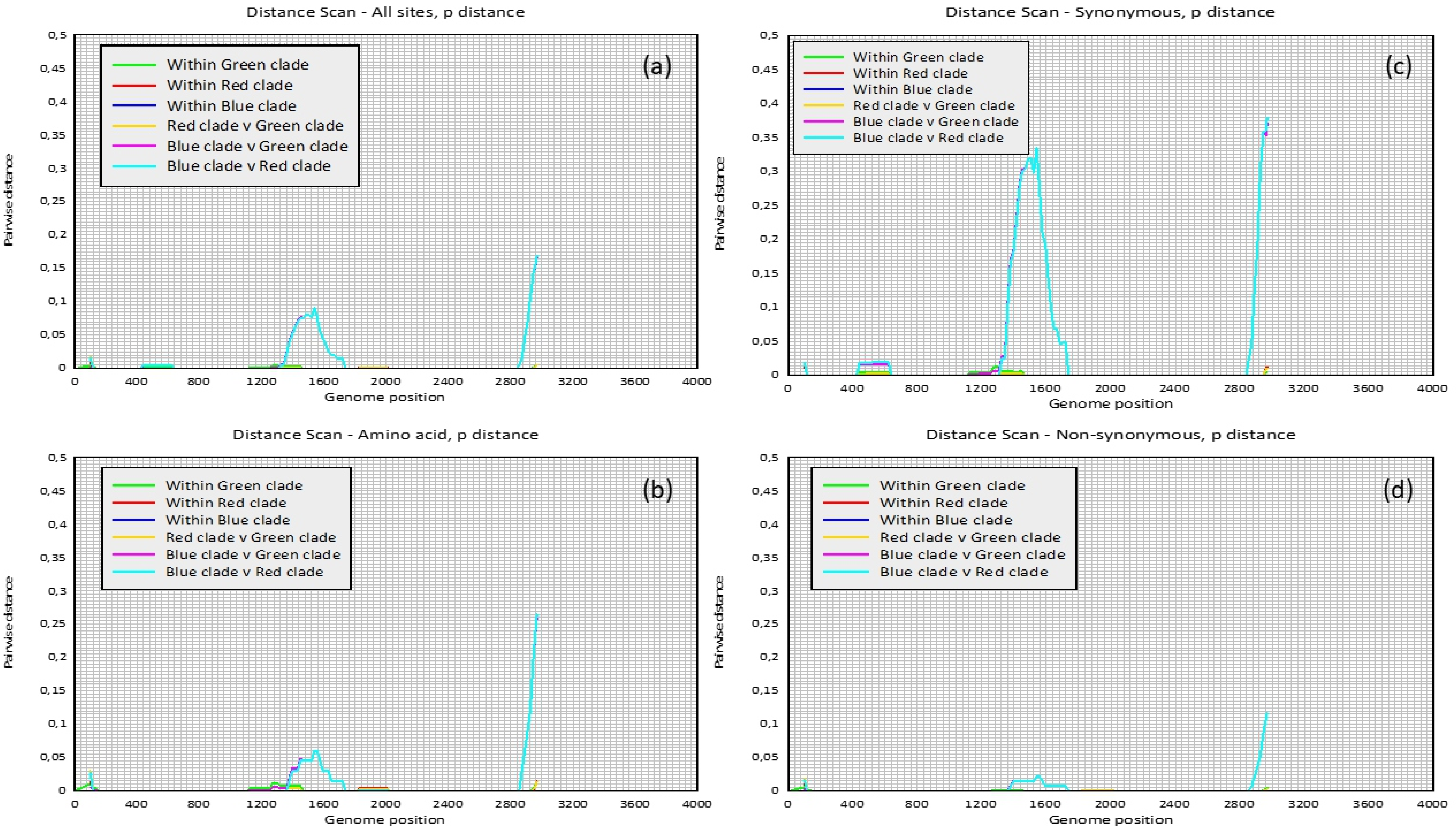
Distance Scan - All sites, p distance
(a)
Within Green clade
Within Red clade
Within Blue clade
Red clade v Green clade
Blue clade v Green clade
Blue clade v Red clade
Pairwise distance
Genome position
Distance Scan - Synonymous, p distance
(c)
Distance Scan - Amino acid, p distance
(b)
Distance Scan - Non-synonymous, p distance
(d)
