# Supplementary material for: Polymorphism analysis of the apxIA gene of Actinobacillus pleuropneumoniae serovar 5 isolated in swine herds from Brazil
Source: PLoS One. 2018 Dec 18;13(12):e0208789. doi: 10.1371/journal.pone.0208789 (PMC6298653; doi:10.1371/journal.pone.0208789)
Supplement: S1 Table — Sequences are divided between the clades green (A), red (B) and blue (C), separated by the number of sequences for each H1—H14 haplotype. Sequences related to the GenBank database are divided between the clades according to the separation made by phylogenetic analyzes. (PDF) [file pone.0208789.s003.pdf]

Supplementary Table 1. The complete list of *apxIA* gene sequences separated by clades according to phylogenetic relationships.

Clade Green (A)	Clade Red (B)	Clade Blue (C)
KY468982 (H1): 33 sequences<br><br>1003_SC_2011<br>1007_SC_2011<br>1015_SC_2011<br>1032_MG_2011<br>1034_SC_2011<br>1036_SC_2011<br>1072_SC_2011<br>1075_SC_2011<br>1078_SC_2011<br>1085_SC_2011<br>1100_SC_2011<br>1119_SC_2011<br>1132_SC_2011<br>426_SC_2006<br>431_SC_2006<br>643_SC_2008<br>674_SC_2008<br>680_SC_2008<br>700_SC_2008<br>725_RS_2009<br>749_PR_2009<br>758_SC_2009<br>766_SC_2009<br>767_RS_2009<br>770_SC_2009<br>792_MS_2009<br>848_MS_2010<br>888_MG_2010<br>915_SC_2010<br>919_SC_2010<br>920_SC_2010<br>946_MT_2010<br>999_SC_2011	KY468984 (H3): 12 sequences<br><br>1061_SC_2011<br>439_SP_2006<br>509_SC_2007<br>510_SC_2007<br>523_SC_2007<br>524_SC_2007<br>760_SC_2009<br>761_SC_2009<br>891_SC_2010<br>893_SC_2010<br>912_SC_2010<br>914_SC_2010	ADOK00000000.1 (56153, serovar 11): 1 sequence
KY468983 (H2): 1 sequence<br><br>430_SC_2006	KY468985 (H4): 1 sequence<br><br>449_ES_2006	ADOI00000000.1 (CVJ13261, serovar 9): 1 sequence

KY468986 (H5): 1 sequence<br><br>461_SP_2006	KY468988 (H7): 1 sequence<br><br>511_SC_2007	NZ_CP029003.1 (4074, serovar 1): 1 sequence
KY468987 (H6): 3 sequences<br><br>491_PR_2007<br>745_SC_2009<br>769_SC_2009	KY468990 (H9): 1 sequence<br><br>791_MS_2009	
KY468989 (H8): 1 sequence<br><br>672_SC_2008	KY468991 (H10): 1 sequence<br><br>857_SC_2010	
KY468995 (H14): 1 sequence<br><br>928_SC_2010	KY468994 (H13): 1 sequence<br>867_RS_2010	
ADOJ00000000 (D13039, serovar 10): 1 sequence	KY468992 (H11): 2 sequences<br><br>861_SC_2010<br>913_SC_2010	
NC_009053.1 (L20, serovar 5b): 1 sequence	KY468993 (H12): 1 sequence<br><br>862_SC_2010	
	KY468990 (H9): 1 sequence<br><br>791_MS_2009	

Sequences are divided between the clades green (A), red (B) and blue (C), separated by the number of sequences for each H1 - H14 haplotype. Sequences related to the GenBank database are divided between the clades according to the separation made by phylogenetic analyzes.
