# Supplementary material for: Polymorphism analysis of the apxIA gene of Actinobacillus pleuropneumoniae serovar 5 isolated in swine herds from Brazil
Source: PLoS One. 2018 Dec 18;13(12):e0208789. doi: 10.1371/journal.pone.0208789 (PMC6298653; doi:10.1371/journal.pone.0208789)
Supplement: S2 Table — a Year in which the isolate was collected;b Property in which the sample was collected;c Identification of isolates in laboratory (isolates were shortened in two digits after the dash);d Groups of haplotypes;e Brazilian state of origin of the isolates that determine the haplotype;f Phylogenetic clade determined in the consensus tree. (PDF) [file pone.0208789.s004.pdf]

Supplementary table 2. Sequences that were not cataloged for the breeding system.

Systems breeding swine	Years [a]	Swine breeders [b]	Isolates [c]	Haplotypes [d]	States [e]	Phylogenetic clade [f]
Non-cataloged systems.	2011	no information	1003	H1	SC	green
	2011	no information	1007	H1	SC	
	2011	no information	1015	H1	SC	
	2011	no information	1032	H1	SC	
	2011	no information	1032	H1	MG	
	2011	no information	1034	H1	SC	
	2011	no information	1036	H1	SC	
	2011	no information	1072	H1	SC	
	2011	no information	1075	H1	SC	
	2011	no information	1078	H1	SC	
	2011	no information	1085	H1	SC	
	2011	no information	1100	H1	SC	
	2011	no information	1119	H1	SC	
	2009	no information	749	H1	PR	
	2009	no information	792	H1	MS	
	2010	no information	848	H1	MS	
	2010	no information	946	H1	MT	
	2011	no information	999	H1	SC	
	2011	no information	1061	H3	SC	red

[a] Year in which the isolate was collected; [b] Property in which the sample was collected; [c] Identification of isolates in laboratory (isolates were shortened in two digits after the dash); [d] Groups of haplotypes; [e] Brazilian state of origin of the isolates that determine the haplotype; [f] Phylogenetic clade determined in the consensus tree.
