# Supplementary material for: Polymorphism analysis of the apxIA gene of Actinobacillus pleuropneumoniae serovar 5 isolated in swine herds from Brazil
Source: PLoS One. 2018 Dec 18;13(12):e0208789. doi: 10.1371/journal.pone.0208789 (PMC6298653; doi:10.1371/journal.pone.0208789)
Supplement: S3 Table — α = Synonymous substitutions rate at a site. β+ = Non-Synonymous substitutions rate at a site. [α<β] = Posterior probability of positive selection at a site. [α>β] = Posterior probability of negative selection at a site. Significance values (p-value/Posterior probability) are in bold. (PDF) [file pone.0208789.s005.pdf]

Supplementary table 3. Table reporting sites detected under statistically significant diversifying selection positive and negative (gray shadow) by three different methods.

MEME				FEL			FUBAR			
Codon	α	β+	p-value	α	β+	p-value	α	β+	Prob [α<β]	Prob [α>β]
3	0.00	696.72	**0.00**	0.00	130.102	**0.039**	5.372	43.860	**0.944**	0.010
1020	0.00	272.24	**0.02**							
508				78.322	0.00	**0.042**				
474				99.317	0.00	**0.043**	24.325	2.270	0.071	**0.904**
996							23.698	2.044	0.066	**0.909**
1000							21.590	1.394	0.067	**0.911**
543							21.624	1.673	0.071	**0.905**
499							21.823	1.808	0.071	**0.903**
983							21.169	1.685	0.073	**0.902**
993							21.437	1.753	0.073	**0.902**
180							21.054	1.645	0.073	**0.902**
487							21.079	1.684	0.074	**0.902**
1005							21.660	1.808	0.074	**0.901**
1008							21.660	1.808	0.074	**0.901**

α = Synonymous substitutions rate at a site.

β^{+} = Non-Synonymous substitutions rate at a site.

[α<β] = Posterior probability of positive selection at a site.

[α>β] = Posterior probability of negative selection at a site.

Significance values (p-value/Posterior probability) are in bold.
